# Combination of cardiac and thoracic pump theories in rodent cardiopulmonary resuscitation: a new method of three-side chest compression

**DOI:** 10.1186/s40635-019-0275-9

**Published:** 2019-12-02

**Authors:** Yu Okuma, Koichiro Shinozaki, Tsukasa Yagi, Kota Saeki, Tai Yin, Junhwan Kim, Lance B. Becker

**Affiliations:** 1 The Feinstein Institute for Medical Research, Northwell Health System, 350 Community Dr. Manhasset, Manhasset, NY 11030 USA; 2 Department of Emergency Medicine, North Shore University Hospital/Long Island Jewish Medical Center, Northwell Health System, Manhasset, NY USA; 3 Nihon Kohden Innovation Center, Cambridge, MA USA

**Keywords:** Cardiac arrest, 3-Side chest compression, High-quality cardiopulmonary resuscitation, Return of spontaneous circulation

## Abstract

**Background:**

High-quality cardiopulmonary resuscitation (HQ-CPR) is of paramount importance to improve neurological outcomes of cardiac arrest (CA). The purpose of this study was to evaluate chest compression methods by combining two theories: cardiac and thoracic pumps.

**Methods:**

Male Sprague-Dawley rats were used. Three types of chest compression methods were studied. The 1-side method was performed vertically with 2 fingers over the sternum. The 2-side method was performed horizontally with 2 fingers, bilaterally squeezing the chest wall. The 3-side method combined the 1-side and the 2-side methods. Rats underwent 10 min of asphyxial CA. We examined ROSC rates, the left ventricular functions, several arterial pressures, intrathoracic pressure, and brain tissue oxygen.

**Results:**

The 3-side group achieved 100% return of spontaneous circulation (ROSC) from asphyxial CA, while the 1-side group and 2-side group achieved 80% and 60% ROSC, respectively. Three-side chest compression significantly shortened the time for ROSC among the groups (1-side, 105 ± 36.0; 2-side, 141 ± 21.7; 3-side, 57.8 ± 12.3 s, respectively, *P* < 0.05). Three-side significantly increased the intrathoracic pressure (esophagus, 7.6 ± 1.9, 7.3 ± 2.8, vs. 12.7 ± 2.2; mmHg, *P* < 0.01), the cardiac stroke volume (the ratio of the baseline 1.2 ± 0.6, 1.3 ± 0.1, vs. 2.1 ± 0.6, *P* < 0.05), and the common carotid arterial pressure (subtracted by femoral arterial pressure 4.0 ± 2.5, 0.3 ± 1.6, vs. 8.4 ± 2.6; mmHg, *P* < 0.01). Three-side significantly increased the brain tissue oxygen (the ratio of baseline 1.4±0.1, 1.3±0.2, vs. 1.6 ± 0.04, *P* < 0.05).

**Conclusions:**

These results suggest that increased intrathoracic pressure by 3-side CPR improves the cardiac output, which may in turn help brain oxygenation during CPR.

## Background

Cardiac arrest (CA) is a significant public health issue worldwide, resulting in many deaths or permanent neurological disabilities [1]. High-quality cardiopulmonary resuscitation (HQ-CPR) is of paramount importance to improve survival from CA [2]. Chest compression is an essential component of CPR [3], yet the mechanism has not been clearly described.

Two different mechanisms of chest compression have been proposed: cardiac pump theory and thoracic pump theory [4–7]. Cardiac pump refers to a pump delivering the blood by directly squeezing the heart between the spine and the sternum, while the thoracic pump theory relates the heart to a passive conduit in which blood is forced away from the thorax when the intrathoracic pressure exceeds extravascular pressure during chest compression.

These competing concepts of whether the heart is a pump or a conduit have caused much controversy. The mechanism of chest compression is often explained by the lung pump theory, in which the heart works as a part of the larger pumping system with the lungs [4, 8]. Rudikoff [7] measured pressures in “the heart pump” in dogs, including the left ventricle, aorta, right atrium, and pulmonary artery, which were identical and equal to the intrathoracic pressure estimated by an esophageal balloon catheter. They postulated the thoracic pump theory in CPR. Although the cardiac pump [6] seems an essential part of supporting the mechanism of CPR, these theories may solely or additionally contribute to HQ-CPR and subsequently aid to improve survival from CA.

The present investigation was undertaken to determine the extent, if any, to which the intrathoracic pressure during chest compression influences the quality of CPR. We developed a new method, termed three-side chest compression, in an attempt to increase the intrathoracic pressure in a rodent CPR model. This novel technique introduces horizontal squeezing of the chest wall, bilaterally, in addition to the traditional sternal compression. To test this new technique, we compared esophageal pressure to both the arterial pressures and the left ventricular pressure during CPR. To the best of our knowledge, this is the first study introducing a way to increase intrathoracic pressure in a rodent CPR model. Furthermore, the effect of our new technique on the successful rate of spontaneous circulation recovery was demonstrated in this study.

## Methods

### Animal preparation

We performed all procedures according to the previously described protocol [9]. Adult male Sprague-Dawley rats (450–550 g, Charles River Laboratories, Wilmington, MA, USA) were anesthetized with 4% isoflurane (Isosthesia, Butler-Schein AHS, Dublin, OH, USA) and intubated by a 14-gauge plastic catheter (Surflo, Terumo Medical Corporation, Somerset, NJ, USA). The rats underwent mechanical ventilation (Ventilator Model 683, Harvard Apparatus, Holliston, MA, USA). We fixed a minute ventilation volume at 180 mL/min at a respiratory rate of 45 breaths per minute (tidal volume: 8–9 ml/kg). The carbon dioxide (CO_2_) was continuously measured inline in the exhalation branch of the ventilator circuit by using an expiratory CO_2_ gas monitor (OLG-2800, Nihon Kohden Corp., Tokyo, Japan) with a CO_2_ sensor (TG-970P, Nihon Kohden Corp., Tokyo, Japan) and airway ETC adapter (YG-211T, Nihon Kohden Corp., Tokyo, Japan). The end-tidal CO_2_ (EtCO_2_) values were monitored within a range of 30–45 mm Hg during preparation. Under anesthesia with isoflurane 2% and a fraction of inspired O_2_ (FIO_2_) of 0.3, a core body temperature probe (T-type thermocouple probes, ADInstruments, Colorado Springs, CO, USA) was placed in the esophagus and the temperature was maintained at 36.5 ± 1.0 °C during the surgical procedure. We inserted a sterile polyethylene-50 catheter in the left femoral artery (FA) for continuous arterial pressure monitoring (MLT844, ADInstruments; Bridge Amplifier ML221, ADInstruments, Colorado Springs, CO, USA). Another polyethylene-50 catheter was cannulated from the left femoral vein and the tip was advanced 9 cm from the insertion site in order to monitor the pressure of the inferior vena cava nearby the right atrium as the central venous pressure (CVP) (MLT844, ADInstruments; Bridge Amplifier ML221, ADInstruments, Colorado Springs, CO, USA). 150 U of heparin (Heparin, SAGENT Pharmaceuticals, Schaumburg, IL, USA) was given through the CVP catheter during the surgical preparation and the basal data were obtained (preparation time, temperature, heart rate, femoral artery pressure, EtCO_2_, etc). At the end of preparation, blood samples were collected from the arterial line before asphyxia. The pH, the partial pressure of oxygen, the partial pressure of carbon dioxide, lactate, glucose, and hematocrit levels were measured (i-STAT, Abbott Laboratories, Abbott Park, IL, USA)

### Survival study

Animals were assigned to 3 groups at the end of surgery: (1) 10-minute asphyxia arrest treated with 1-side chest compression (*n*=5), (2) 10-min asphyxia arrest treated with 2-side chest compression (*n* = 5), and (3) 10-min asphyxia arrest treated with 3-side chest compression (*n* = 5). After preparation, 2 mg/kg of vecuronium bromide (Hospira, Lake Forest, IL, USA) was slowly given to all animals. The ventilator was turned off to introduce asphyxia to rats. CA generally occurred within 3 to 4 min after asphyxia was initiated. All animals were positioned on the spine position. After the initial 10 min, we restarted mechanical ventilation at an FIO_2_ of 1.0 and performed 3 types of manual CPR. One-side was chest compression vertically performed with 2 fingers over the sternum at a rate of 240 to 300 per minute. The chest compression rate was obtained retrospectively from the record of pressure waveforms of the femoral arterial catheter. Two-side method was performed with 2 fingers horizontally squeezing the chest wall from both sides at the same rate, while 3-side chest compression was underwent with the right hand’s 2 fingers over the sternum in synchrony with left hand’s 2 fingers squeezing the chest wall (Additional file 1). Thirty seconds after the beginning of CPR, a 20-μg/kg bolus of adrenaline was administered through the venous catheter. We defined the return of spontaneous circulation (ROSC) as a systolic blood pressure over 60 mmHg, after which chest compression was discontinued. If we could not obtain ROSC by 5 min from the initiation of CPR, we terminated resuscitation. Mechanical ventilation was discontinued 2 h after CPR, and survival was monitored 10 min after extubation. The left FA pressure and CVP were monitored in all animals. After euthanizing the animals, the position of the central vein catheter tip was verified in all cases and, if not positioned appropriately, the CVP data was omitted accordingly. In an attempt to further elucidate the beneficial effects of 3-side chest compression in a rodent CPR model, we also tested ROSC rates of (1) 14-min asphyxia arrest treated with 3-side chest compression (*n* = 4) and (2) 15-min asphyxia arrest treated with 3-side chest compression (*n* = 4).

### The comparison of arterial pressures and intrathoracic pressure

A set of experiments separated from the survival study was conducted for physiological measurements of three different chest compressions. In this study, we started chest compression 10 min after the induction of asphyxiation without giving adrenaline. No animals obtained ROSC in this experiment. The three sets of chest compressions were alternately tested every 60 s, and CPR was performed for up to 6 min. After the rats were intubated and mechanically ventilated with oxygen-containing isoflurane, a midline cervical incision was performed under local analgesics. The polyethylene-50 catheter was advanced into the right common carotid artery (CCA). The CCA and FA were recorded at the same time from the same animals (*n* = 6). In addition to these experiments, another set of experiments involving measurement of the intra-esophageal pressure, the endotracheal airway pressure, and the left ventricle pressure was conducted (*n* = 6). A pressure transducer catheter (MPC500, Millar Instruments, Houston, TX, USA) was placed in the esophagus and the intra-esophageal pressure was monitored. A pressure probe (MLT844, ADInstruments; Bridge Amplifier ML221, ADInstruments, Colorado Springs, CO, USA) was attached inline in the branch of the ventilator circuit and the endotracheal airway pressure was continuously recorded. The polyethylene-50 catheter was then advanced to the left ventricle from the right common carotid artery via ascending aortic arch. The catheter tip position was verified by monitoring the pressure waveforms.

### Cardiac output

To determine the effect of different chest compressions on cardiac output, a 1.9 Fr pressure-volume (PV) conductance catheter (Model FTE-1912B-8018, Scisense Inc., London, ON, Canada) was inserted into the left ventricular from the right CCA via ascending aortic arch (*n* = 5). After a 10-min asphyxiation, we alternately performed 3 different chest compressions without mechanical ventilation or adrenaline injection. The 3 types of chest compression was applied every 20 s and CPR was continued for up to 5 min. We evaluated the stroke volume using the ADVantage™ system (Scisense Inc., London, ON, Canada).

### Brain tissue oxygen

A quenching oxygen probe (AL300, Ocean Optics, Dunedin, FL, USA) and a fluorometer (NEOFOX-GT, Ocean Optics, Dunedin, FL, USA) were used to measure the oxygen level in the brain tissue during CPR (*n* = 4). The response time of this oxygen sensor is less than 1 s. After rats were intubated and mechanically ventilated with oxygen-containing isoflurane, the body was secured in a prone position. We made a 2-mm bur-hole on the right parietal cortex (3 mm posterior and 3 mm lateral to the bregma). A 20-gauge plastic catheter (Surflo, Terumo Medical Corporation, Somerset, NJ, USA) was inserted into the subdural space and the quenching oxygen probe was advanced through the catheter. After completing the surgical preparation, the animals were repositioned to supine position. After a 10-min asphyxiation, we alternately performed three different chest compressions without administering adrenaline. Each chest compression was performed and switched every 20 s and continued for up to 4 min. The average of 10 s at the last half of the compression cycle was recorded and analyzed as the oxygen level in the brain tissue.

## Statistical analysis

Data were reported as mean and SD. The Fisher exact test for categorical variables and Mann–Whitney *U* test for continuous variables were used. To compare 3 groups, we used a 1-way analysis of variance (ANOVA) with post hoc analysis using the Tukey test. All statistical analyses were performed with EZR (Saitama Medical Center, Jichi Medical University, Saitama, Japan), which was modified version of R (The R Foundation for Statistical Computing, Vienna, Austria) commander designed to add statistical functions frequently used in biostatistics [10]. *P* values less than 0.05 were considered significant.

## Results

### Effect of 3-side chest compression on the thoracic pump

**Fig. 1:**
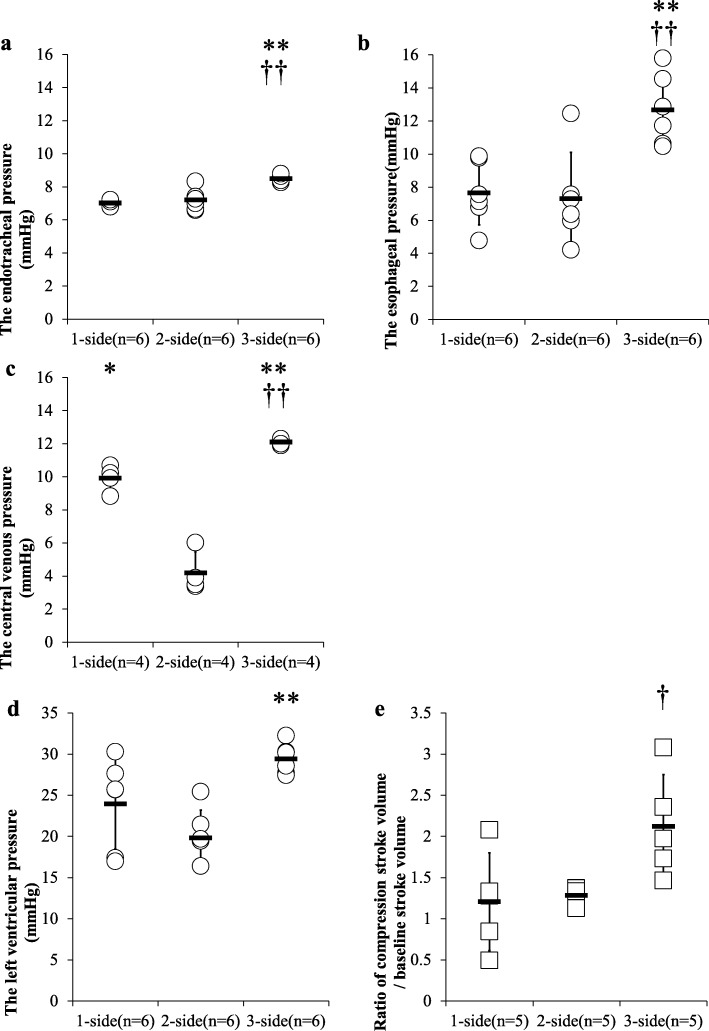
The evaluation related to the intrathoracic pressure during chest compression (**a**–**c**) and the evaluation related to the cardiac output during chest compression (**d**, **e**) **a** This figure shows the endotracheal airway pressure compared among the three groups. The results are shown as mean ± SD. ††*P* < 0.01 compared with the 1-side group. **P < 0.01 compared with the 2-side group. **b** The esophageal pressure. The results are shown as mean ± SD. ††*P* < 0.01 compared with the 1-side group. **P < 0.01 compared with the 2-side group. **c** The central venous pressure. The results are shown as mean ± SD. ††*P* < 0.01 compared with 1-side group. *P < 0.05 compared with the 2-side group. **P < 0.01 compared with the 2-side group. **d** The left ventricular pressure. The results are shown as mean ± SD. **P < 0.01 compared with the 2-side group. **e** The ratio of stroke volume compared with the baseline. The results are shown as mean ± SD. †*P* < 0.05 compared with 1-side group

Figure 1 depicts the effect of 3-side chest compression on the parameters of intrathoracic pressure. Three-side chest compression significantly increased the endotracheal airway pressure (1-side, 2-side, and 3-side: 7.0 ± 0.1, 7.2 ± 0.6, and 8.5 ± 0.2 mmHg, respectively) and the esophageal pressure (1-side, 2-side, and 3-side, 7.6 ± 1.9, 7.3 ± 2.8, and 12.7 ± 2.2 mmHg, respectively) (Fig. 1a, b). Three-side chest compression significantly increased the CVP at 10 s after CPR (1-side, 2-side, and 3-side, 9.9 ± 0.8, 4.2 ± 1.2, and 12.1 ± 0.2 mmHg, respectively) (Fig. 1c). As seen in Additional file 2, there were correlations of the left ventricular pressure with both esophageal and endotracheal pressures (correlation coefficient = 0.589, *P* < 0.05; correlation coefficient = 0.571, *P* < 0.05, respectively).

### Effect of 3-side chest compression on cardiac output

Concerning the left ventricular function, 3-side chest compression significantly increased the pressure (1-side, 2-side, and 3-side, 24.0 ± 5.5, 19.8 ± 3.4, and 29.4 ± 1.8 mmHg, respectively) (Fig. 1d). Three-side also increased the stroke volume (1-side, 2-side, and 3-side, 1.2 ± 0.6, 1.3 ± 0.1, and 2.1 ± 0.6, respectively) (Fig. 1e).

### Effect of 3-side chest compression on ROSC rate

There were no significant differences in the basal characteristics among the groups (Table 1). Animals in the 3-side group achieved 100% (5/5) ROSC rate, while it was 80% (4/5) in the 1-side group and was 60% (3/5) in the 2-side group (*P* = 0.725).

**Table 1:** The basal background including operative time and chest compression rate

	1-side	2-side	3-side	*P* value
	*n* = 5	*n* = 5	*n* = 5	
Weight, g	472 ± 16	467 ± 16	476 ± 17	0.800
Preparation time, minutes	61.4 ± 21.7	60.6 ± 28.7	60.2 ± 31.3	0.998
Chest Compression rate, BPM	275 ± 9	278 ± 11	278 ± 11	0.833
Blood gas	*n* = 5	*n* = 5	*n* = 5	
PH	7.39 ± 0.02	7.37 ± 0.04	7.38 ± 0.04	0.734
PO_2_, mm Hg	103 ± 20	104 ± 38	103 ± 15	0.994
PCO_2_, mm Hg	41.5 ± 6.7	45.8 ± 7.3	42.3 ± 4.8	0.538
Lactate, mmol/L	1.26 ± 0.47	1.19 ± 0.39	1.18 ± 0.29	0.948
Glucose, mg/dL	245 ± 97.2	227 ± 90	223 ± 49	0.904
HCT, %	49 ± 3	48 ± 4	48 ± 2	0.821

Blood samples were obtained from the arterial line before asphyxia (baseline). Values are expressed as mean ± SD

*BPM* beat per minute, *PO*_*2*_ partial pressure of O_2_, *PCO*_*2*_ partial pressure of carbon dioxide, *HCT* hematocrit

### Effect of 3-side chest compression on the quality of CPR

**Fig. 2:**
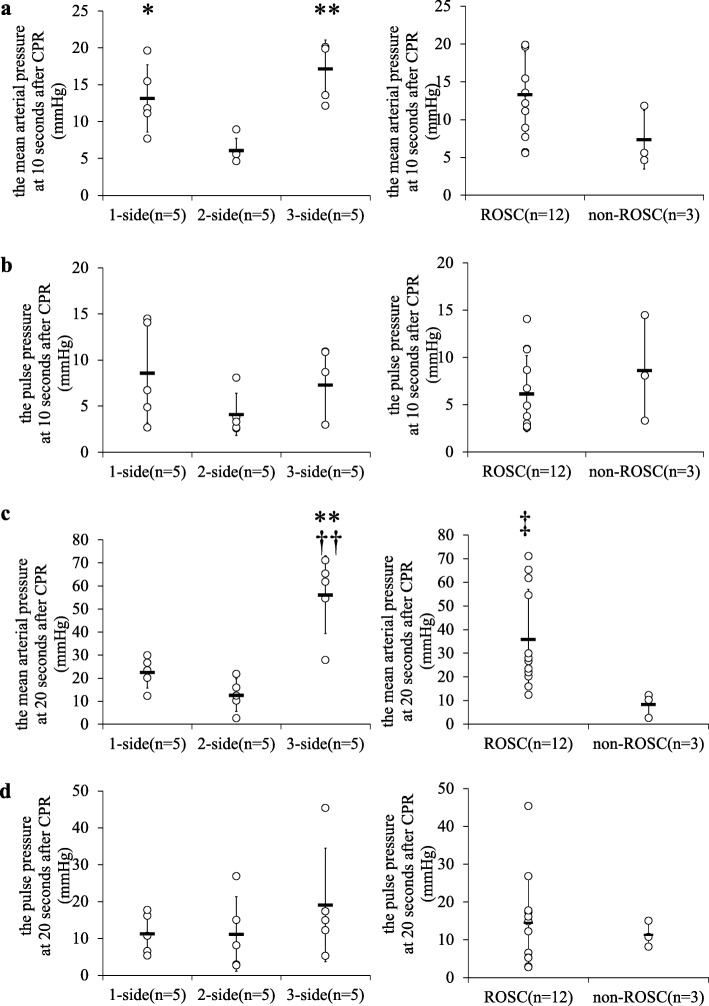
The evaluation of the mean arterial pressures (MAP) and the pulse pressure (PP) at 10 s after CPR and at 20 s after adrenaline injection. **a** This figure shows MAP at 10 s after CPR starting. The results are shown as mean ± SD. *P < 0.05 compared with the 2-side group. **P < 0.01 compared with the 2-side group. **b** PP for 10 s after CPR starting. The results are shown as mean ± SD. **c** MAP for 20 s after adrenaline injection. The results are shown as mean ± SD. ††*P* < 0.01 compared with the 1-side group. **P < 0.01 compared with the 2-side group. ‡*P* < 0.05 compared with the non-return of spontaneous circulation (ROSC) group. **d** PP for 20 s after adrenaline injection. The results are shown as mean ± SD

The hemodynamic parameters of rats were compared. Three-side chest compression significantly increased the mean arterial pressure (MAP) at 10 s after CPR (1-side, 2-side, and 3-side, 13.2 ± 4.6, 6.1 ± 1.6, and 17.1 ± 3.9 mmHg, respectively) and at 20 s after adrenaline injection (1-side, 2-side, and 3-side, 23.6 ± 6.8, 12.7 ± 7.1, and 56.2 ± 16.9 mmHg, respectively). The ROSC group and the non-ROSC group had significant differences in MAP at 20 s after adrenaline injection (ROSC and non-ROSC, 36.0 ± 21.0 and 8.5 ± 5.1 mmHg). On the other hand, the pulse pressure (systolic minus diastolic pressure) showed no significant differences at 10 s after CPR (1-side, 2-side, and 3-side, 8.6 ± 5.4, 4.1 ± 2.3, and 7.3 ± 4.0 mmHg, respectively) and at 20 s after adrenaline injection (1-side, 2-side, and 3-side, 11.4 ± 5.5, 11.2 ± 10.1, and 19.1 ± 15.4 mmHg, respectively). In addition, there were no differences in pulse pressures between the ROSC group and the non-ROSC group (ROSC and non-ROSC, 14.5 ± 12.2, and 11.4 ± 3.5 mmHg) (Fig. 2).

**Fig. 3:**
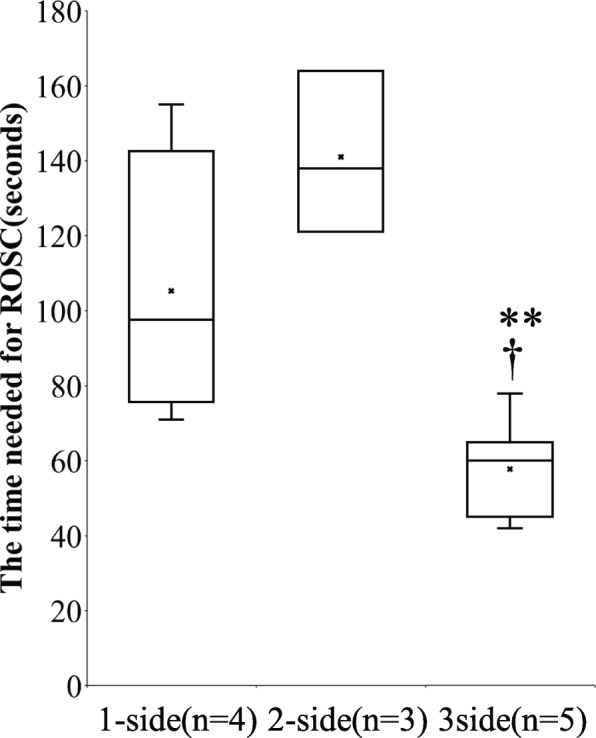
The evaluation of the time required for return of spontaneous circulation (ROSC). The mean is shown as X in the box charts. †*P* < 0.05 compared with the 1-side group. **P < 0.01 compared with the 2-side group

Figure 3 shows the results of time from CPR to ROSC compared among the groups. Three-side chest compression significantly reduced the time needed for ROSC (1-side, 2-side, and 3-side 105 ± 36.0, 141 ± 21.7, and 57.8 ± 12.3 s, respectively).

**Fig. 4:**
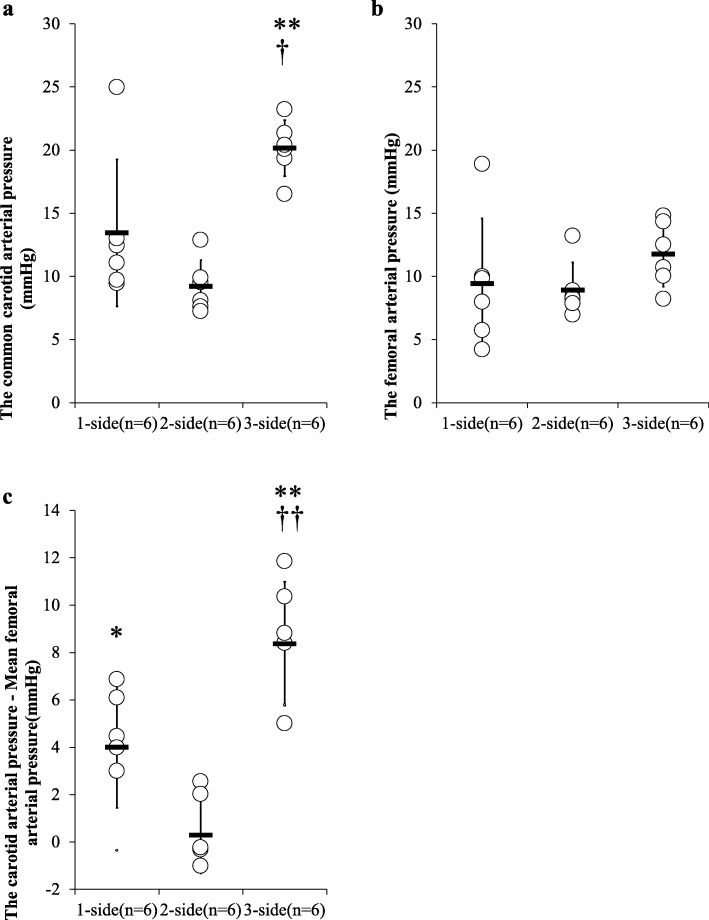
The comparison of the right common carotid arterial (CCA) pressure and the left femoral arterial (FA) pressures at the same time during chest compression. **a** This figure shows the CCA pressure. The results are shown as mean ± SD. †*P* < 0.05 compared with 1-side group. **P < 0.01 compared with the 2-side group. **b** The FA pressure. The results are shown as mean ± SD. **c** The CCA pressure minus FA pressure at the same time. The results are shown as mean ± SD. *P < 0.05 compared with the 2-side group. **P < 0.01 compared with the 2-side group. ††*P* < 0.01 compared with the 1-side group

Figure 4 shows the effect of three different compressions on CCA, FA, and the difference between CCA and FA. Three-side chest compression significantly increased CCA (1-side, 2-side, and 3-side, 13.4 ± 5.8, 9.2 ± 2.1 and 20.2 ± 2.2 mmHg, respectively). On the other hand, there was no significant difference in FA (1-side, 2-side and 3-side, 9.4 ± 5.1, 8.9 ± 2.2, and 11.8 ± 2.6 mmHg, respectively). Three-side chest compression significantly increased the difference of CCA and FA (1-side, 2-side, and 3-side, 4.0 ± 2.5, 0.3 ± 1.6 and 8.4 ± 2.6 mmHg, respectively).

### Effect of 3-side chest compression on brain oxygenation and EtCO_2_

Concerning the tissue oxygen partial pressure of the brain (PbO_2_), 3-side chest compression significantly increased the ratio compared to the baseline (1-side, 2-side, and 3-side, 1.4 ± 0.1, 1.3 ± 0.2 and 1.6 ± 0.04, respectively). Three-side significantly increased EtCO_2_ (1-side, 2-side, and 3-side, 12.4 ± 2.0, 14.3 ± 1.9, and 17.5 ±1.7 mmHg, respectively) (Additional file 3).

### 3-Side chest compression might enable us to make a severer model

In the additional ROSC rate study, animals in the 3-side group achieved 75% (3/4) ROSC rate from 14-min asphyxial CA model, while it was 0% (0/4, *P* = 0.143) in the 15-min asphyxia model.

## Discussion

The present investigation found that the 3-side chest compression increased the intrathoracic pressure during chest compression. In this study, the intrathoracic pressure was estimated from the esophageal pressure and the endo-tracheal pressure—as discussed in previous studies [7, 11, 12]. It is highly plausible that the increased intrathoracic pressure subsequently improved the quality of CPR in our asphyxia CA rat model. The quality of CPR was evaluated by measuring the FA pressure, the CCA pressure, the difference between CCA and FA pressures, the left ventricle pressure, and the cardiac output. These findings may increase the brain oxygenation and EtCO_2_, which are important indicators of the quality of CPR [2].

We developed a new/unique CPR technique, 3-side chest compression, in an attempt to increase the intrathoracic pressure. To the best of our knowledge, this is the first study to introduce chest compression that successfully increased intrathoracic pressure in a rodent CPR model. Previous reports showed that a 10-min asphyxia model of CA was one of the most successful and controllable models. The successful ROSC rate was reported as 65–88% [9, 13, 14]. Our method reached a ROSC rate of 75% from prolonged asphyxia CA (maximum 14 min). Except for a neonatal model, the asphyxia time for a CPR rodent model is commonly up to 12 min [13]. This result suggested that 3-side chest compression would enable researchers to use a more severe CPR model in rats.

In this study, we evaluated esophageal pressure, endotracheal airway pressure, and CVP to estimate the intrathoracic pressure and our results strongly supported that 3-side chest compression increased the intrathoracic pressure. All of our measurement techniques are reproducible and showed statistical significance; however, the most meaningful change was observed in the esophageal pressure. Therefore, we recommend measuring the esophageal pressure to estimate the intrathoracic pressure in a rodent CA model.

Convertino [11] and Lurie [12] introduced the use of the inspiratory impedance threshold valve during CPR in a porcine CA model and investigated the effect of regulating the endotracheal pressure along with intrathoracic pressure. The negative pressure of intrathoracic pressure during relaxation of chest compressions can maximize the effect of the thoracic pump [15]. On the other hand, other investigator looking at a positive entotracheal airway pressure during all the CPR cycle [16] demonstrated that no negative pressure was generated during the decompression phase of CPR with some negative effect on hemodynamic. However, it has been widely accepted that simultaneous chest compression and ventilation at high airway pressure improves the systemic arterial pressure in humans [17, 18] and experimental models [19]. An open airway limits the effect of the thoracic pump [8] as the pressure measured in the airway becomes equal to the atmospheric pressure. In our experimental setting, the ventilation system was used with a closed circuit. There was no synchronization of CPR with the ventilation cycles. The 3-side chest compression showed the highest airway pressure among the groups. The same trend in negative pressure was observed during the decompression phase of CPR (Additional file 4). However, the 3-side chest compression had the same or moderately lower left ventricle pressure during the decompression of CPR (Additional file 4). These results suggest that the 3-side might increase the intrathoracic pressure and it, in turn, yield a subsequent increase of the endotracheal airway pressure: however, due to no synchronization of the decompression in CPR with ventilation, the relaxation effect was not clearly seen from our data. Future studies may wish to investigate and develop a method to maximize the relaxation effect in a rodent CPR model.

The competing concepts as to whether the heart is a pump or a conduit have caused much controversy. As a result, the mechanism of chest compression is often explained by the lung pump theory, in which the heart works as a part of the larger pumping system with the lungs [4, 8]. Our work was not conducted to separately investigate this point. Although the cardiac pump [6] seems an essential part of supporting the mechanism of CPR, both cardiac and thoracic pumps may synergistically contribute to HQ-CPR and subsequently improve survival from CA. It is inferred that both theories need to work together to maximize its benefit of CPR.

Hemodynamic compromise deteriorates the function of vital organs such as the brain [20, 21]. Since 3-side chest compression increased the effect of thoracic pump and the CCA pressure, this might contribute to increased oxygenation of the brain during CPR. Monitoring brain tissue oxygen, such as PbO_2_ and rSO_2_, is an easy-to-use method and reflects the quality of CPR [22, 23]. However, we did not evaluate the relationship between intracranial pressure and intrathoracic pressure during chest compression mainly due to technical difficulties in a small rodent model. It has not been elucidated how an increase of intrathoracic pressure affect intracranial pressure, which may have a deleterious effect on brain oxygenation. Further study with larger gyrencephalic species (swine, non-human primate etc.) may wish to be conducted to investigate the relationship between the intracranial and intrathoracic pressures. EtCO_2_ is used as an indicator of the quality of CPR. As higher EtCO_2_ levels are observed, better perfusion is supported by CPR, increasing the chances of survival [24, 25]. Therefore, our findings of increased PbO_2_ and EtCO_2_ may support that 3-side chest compression led to the better long-term outcomes in CA rats.

The study results were shown from our high-fidelity CA rodent model. Our concept of horizontally compressing the bilateral chest wall is clinically applicable. However, it is not simple to apply our method to other species and humans because of the variability in chest compression frequency and depth with manual CPR, the difference in morphology and physiology. Higher CPR rate in a rodent model significantly limits time for the cardiac relaxation during CPR. Hwang [26, 27] introduced a new/interesting simultaneous sternothoracic cardiopulmonary resuscitation device and published their preliminary data in both mongrel dogs and patients. Likewise, a clinical device development that applies our concepts into humans is feasible and it is in high demand for improving survival from CA.

The current study has several limitations. First, we were not able to completely eliminate the potential selection bias. However, this study was conducted in a randomized manner and there were no significant differences in background data, including operative time and chest compressive rate. Therefore, we considered its possibility was minimized. Second, long-term assessment may be necessary. A longer observation time was associated with more confounding factors, so we focused on the acute phase in this study. Further studies may wish to investigate the effect of 3-side chest compression on long-term outcome.

## Conclusions

The 3-side method increased intrathoracic pressure and likely stabilized cardiac hemodynamic status, which might be beneficial to the brain. This method could become the new standard for rodent CPR, and it would allow for longer periods of ischemia and increased intra-arrest injury, which would have important implications for more realistic modeling of human cardiac arrest.

## Supplementary information

**Additional file 1.** The explainer video how to perform each chest compression.

**Additional file 2. **The correlations of the left ventricular pressure with esophageal and endotracheal pressures. The correlation coefficient of the left ventricular pressure with the esophageal pressure was 0.589 (95% CI: 0.168 to 0.828; *p* < 0.05). The correlation coefficient of the left ventricular pressure with the endotracheal pressures was 0.5713 (95% CI: 0.143 to 0.820; *p* < 0.05).

**Additional file 3. **The brain tissue oxygen partial pressure (PbtO2) and end-tidal CO2 (EtCO2) during chest compression before the return of spontaneous circulation (ROSC). a This figure shows the PbtO2 and EtCO2 in the order of time scales after the start of chest compression. The square indicates the 3-side group. The rhombus indicates the 2-side group. The triangle indicates the 1-side group. And the gray circles indicate EtCO2. b The ratio of PbtO2 compared with the start of chest compression. The results are shown as mean ± SD. * *P* < 0.05 compared with 2-side group. c EtCO2. The results are the mean ± SD. † *P* < 0.05 compared with 1-side group.

**Additional file 4. **The evaluation of the left ventricular and airway pressures during the decompression phase of CPR. a This figure shows the left ventricular pressure compared among the three groups. The results are shown as mean ± SD. † *P* < 0.05 compared with 1-side group. b The airway pressure. The results are shown as mean ± SD. ††*P* < 0.01 compared with the 1-side group. * *P* < 0.05 compared with the 2-side group.

## Data Availability

The datasets used and/or analyzed during the current study are available from the corresponding author on reasonable request.

## Abbreviations

BPM: Beat per minute
CA: Cardiac arrest
CCA: Common carotid artery
CPR: Cardiopulmonary resuscitation
CVP: Central venous pressure
EtCO_2_: End-tidal carbon dioxide
FA: Femoral artery
FIO_2_: Fraction of inspired oxygen
Fr: French
HCT: Hematocrit
HQ-CPR: High-quality cardiopulmonary resuscitation
MAP: Mean arterial pressure
PbO_2_: Tissue oxygen partial pressure of the brain
PCO_2_: Partial pressure of carbon dioxide
PO_2_: Partial pressure of oxygen
PV: Pressure-volume
ROSC: Return of spontaneous circulation
SD: Standard deviation
